# Too old to be included: age diversity statements foster diversity yet fall short on inclusion

**DOI:** 10.3389/fpsyg.2024.1303224

**Published:** 2024-07-08

**Authors:** Oriana De Saint Priest, Franciska Krings, Claudia Toma

**Affiliations:** ^1^Department of Organizational Behaviour & Human Resources, Lee Kong Chian School of Business, Singapore Management University, Singapore, Singapore; ^2^Department of Organizational Behavior, Faculty of Business and Economics, Université de Lausanne, Lausanne, Switzerland; ^3^Solvay Brussels School of Economics and Management, Université Libre de Bruxelles, Brussels, Belgium

**Keywords:** diversity and inclusion, workplace diversity, work teams, diversity policies, diversity statements, age, age diversity, age discrimination

## Abstract

Older employees often face discrimination and exclusion from work teams. In two scenario studies, we tested the impact of age diversity statements on the representation and inclusion of older employees in teams. In Study 1 (*N* = 304), participants had to create a team and were either exposed to a diversity statement or not before selecting two teammates from a list of four differing in age and gender. Then, we measured participants’ inclusive behavioral intentions towards a new, older member joining this team. Age diversity statements increased the representation but not the inclusion of older individuals in teams. In Study 2 (*N* = 518), we further manipulated the content of the statement (diversity or diversity and inclusion) and the organizational motive (reputation or change). We replicated the effects of diversity statements on representation. Moreover, statements also increased certain inclusive behaviors, but only when they targeted diversity and inclusion and reflected an organizational commitment to change. Taken together, these results suggest that age diversity statements foster diversity, yet fail to systematically increase inclusion.

## Introduction

Many countries currently face an increasing proportion of older people in their working population due to longer life expectancies and delayed retirements. In this context, establishing equal opportunities for younger and older employees is crucial. However, older employees often face age-based discrimination at work (e.g., for promotion and training opportunities, Gordon and Arvey, 2004; see also Bal et al., 2011) and experience exclusion from work groups and teams (Marchiondo, 2022). As a result, organizations use a variety of initiatives to foster greater age diversity and inclusion in organizational groups. One of the most frequently used initiatives consists in the implementation of diversity statements (Wang et al., 2023), reflecting the organization’s commitment to age diversity and inclusion by promoting the fair treatment of mature workers. These statements aim to advocate the unbiased treatment of older workers by addressing the social identity processes that lead to negative stereotyping and exclusion (Shore et al., 2011; Parker and Andrei, 2020).

In this paper, we examine whether age diversity statements successfully increase the representation and inclusion of older employees. While some studies suggest that diversity statements could help organizations achieve more diversity and inclusion on various dimensions, others raise doubts about this possibility. Shedding light on the effects of diversity statements is crucial because diversity statements play a key role in determining how diversity is regarded by internal stakeholders (employees) as well as external ones (investors, government, community). Organizations contribute to socially constructing diversity in terms of age, gender, and other attributes by positioning it either as a liability in need of protection or as a source of competitive advantage (Singh and Point, 2006). Understanding whether diversity statements help advance diversity and inclusion or trigger paradoxical and unwanted effects is therefore important both for theory and practice.

From a signaling perspective, organizations use age diversity statements to communicate their values and encourage pro-diversity behaviors among employees (Dover et al., 2020; De Saint Priest and Krings, 2024). By communicating their priorities, they signal what is valued and desired, or, in other words, their expectations regarding employee behavior aligned with organizational diversity goals (Ostroff and Bowen, 2000; Leslie, 2019). Indeed, age diversity statements often explicitly emphasize the value of working in age-diverse groups with a fair mix of older and younger employees while promoting the inclusion of mature workers in teams (Johnson et al., 2020). By doing so, they communicate the desired behavior (i.e., to represent and include older individuals in teams), counteracting processes that may lead to age bias and further exclusion of older employees (Pless and Maak, 2004; Avery et al., 2007; Dover et al., 2020; Parker and Andrei, 2020). Indeed, previous research has demonstrated the positive effects they have on promoting age diversity within teams, even in situations where the stakes are high (De Saint Priest and Krings, 2024). Studies on race and gender diversity statements demonstrate their effects on other dimensions such as organizational perceptions (e.g., attractiveness) and performance of minority individuals (Purdie-Vaughns et al., 2008; Avery et al., 2013; Jansen et al., 2015; Wilton et al., 2015; Apfelbaum et al., 2016; Windscheid et al., 2016).

However, people may also become skeptical about the effectiveness of diversity statements and look for proof of sincerity and progress (Windscheid et al., 2016; Wilton et al., 2020). They tend to evaluate diversity statements as truthful when there is evidence of diversity progress but perceive them as a form of “diversity washing” when diversity progress is lacking (Purdie-Vaughns et al., 2008). Consequences of such forms of counterfeit diversity (Kroeper et al., 2022) or diversity dishonesty (Wilton et al., 2020) include perceptions of the organization as hypocritical and less legitimate as well as decreased inclusion and commitment (De Cock et al., 2024). Diversity statements can also lead to identity threat that undermines performance-related outcomes of individuals belonging to both a racial and gender minority group when these statements are interpreted as institutional pressure to assimilate into the dominant group (Wilton et al., 2015).

Despite these controversial effects, age diversity statements may still be useful in promoting diversity and inclusion in teams for several reasons. First, diversity fatigue and diversity washing effects were mainly found for gender diversity and diversity in general, while the effects of age-specific diversity statements are less researched. Unlike measures targeting gender or race, all workers will eventually benefit from age diversity initiatives as they grow older. Second, research shows that people are sensitive to age diversity statements and act in accordance with its values, without these statements causing unintended side effects (De Saint Priest and Krings, 2024). Emphasizing age diversity may increase workers’ perceived person-organization fit by promoting organization-based respect since workers anticipate benefiting from fair treatment inside the organization as they age (Ihme et al., 2016). Thus, we propose that age diversity statements promote behaviors that increase the representation and inclusion of older individuals. We therefore hypothesize that age diversity statements will increase the representation (Hypothesis 1) and inclusion (Hypothesis 2) of older individuals in teams so that teams become more age-diverse and inclusive. We tested these hypotheses in two scenario studies, where we evaluated the impact of age diversity statements on people’s willingness to choose older teammates and further include another older person in their team.

## Study 1

### Method

#### Participants

We recruited 304 U.S and U.K residents using the Prolific platform. Participants were paid £1.50 for a study we expected to take 10 min, corresponding to an hourly wage of £9. After excluding respondents who failed the attention check (*n* = 11) and who did not indicate their gender (*n* = 4), the final sample consisted of 289 participants (mean age 40.16, *SD* = 13.69, 49.1% men). The majority were employed (53.3% full-time, 21.1% part-time), and the remaining 25.6% were unemployed.

#### Procedure

Participants were randomly allocated to one of two conditions (age diversity statement: yes or no). In both conditions, they read a business scenario in which they were solely responsible for a challenging project. Participants were informed that they had to create a project team of three persons to complete the project by selecting two teammates out of a list of four, who were all described as equally good and reliable. The four potential teammates varied with respect to age and gender and were described as follows: “The four collaborators currently available to work on your project are Robert, David, Rebecca, and Jennifer. You have met all four of them and got an idea of what they are like. This is what you know about their age: Robert is 60 years old, Rebecca is 62 years old, David is 27 years old, and Jennifer is 29 years old.” These four names are among the most frequent in the U.S. (Social Security Administration, 2024).

Before selecting their teammates, participants in the age diversity statement condition were shown the age diversity statement. In line with previous research (De Saint Priest and Krings, 2024), the statement read: “The company you work for cherishes age diversity in teams. Given the current demographic aging, it is very important to encourage work with older employees. Thus, the company encourages you to work in teams where older employees are well represented.” This statement appeared on a separate screen. Participants in the other condition were not shown an age diversity statement. After choosing their teammates, participants were informed that another colleague was joining their team (Ronnie, 63 years old). Participants then indicated how much they intended to engage in inclusive behaviors toward this new team member. At the end of the survey, they answered some questions about their demographic background.

#### Main variables

To measure the representation of older individuals in teams, we counted the number of older teammates chosen by the participant, which could range from 0 to 2. To measure the inclusion of older individuals in teams, we assessed behavioral intentions towards the additional older team member, avoiding pure spillover effects towards the previously chosen older team members. We used adapted versions of four scales covering different aspects of team inclusion: group involvement (6 items; e.g., “I will make him/her feel part of informal discussions in the workgroup”), influence in decision making (4 items, e.g., “I will make sure that s/he will have a say in the way work is performed”), group belonging (3 items, e.g., “I will give him/her the feeling that s/he belongs”) and authenticity (4 items, e.g., “I allow him/her to express him/herself the way s/he is”). Group involvement and influence in decision-making are subscales of the Inclusion–Exclusion scale (MBIE) by Mor-Barak and Cherin (1998), while group belonging and authenticity are subscales of the Perceived Group Inclusion scale (PGIS) by Jansen et al. (2014). Participants indicated how likely they were to engage in certain behaviors towards the older team member on a 6-point likelihood response scale for the MBIE subscales and a 5-point likelihood response scale for the PGIS subscales. Additionally, we measured inclusion through resource sharing by asking participants to distribute a €100 team bonus between their three teammates, including the new older teammate. The order of appearance of the different inclusion measures was randomized.

#### Control variables

We controlled for participants’ gender (1 = *male*, 2 = *female*), age, ethnicity (0 = *non-White/Caucasian*, 1 = *White/Caucasian*), employment status (0 = *unemployed*, 1 = *employed*), and experience of work in age-diverse teams (“How much experience do you have working in teams that are mixed, in terms of age?”; 5-point scale, 0 = *none*, 4 = *a lot*).^1^

### Results

Correlations, descriptive statistics, and reliabilities for the study variables are presented in Table 1. To test Hypothesis 1, we conducted an analysis of covariance (ANCOVA) with the number of selected older teammates as an outcome, age diversity statement condition as a predictor, and participants’ age, gender, ethnicity, employment status, and experience of work in age-diverse teams as controls. Descriptive statistics per condition and results are displayed in Table 2 (upper half). As expected, participants were more likely to select an older individual for their team with an age diversity statement in place compared to when there was no age diversity statement.

**Table 1:** Study 1: means, standard deviations, reliabilities and correlations between study variables.

		Mean (SD)	1	2	3	4	5	6	7	8	9	10	11
1.	Div. statement	0.50 (0.50)	–										
2.	Nr older	1.03 (0.47)	0.12*	–									
3.	PGIS member	4.69 (0.50)	−0.01	0.12	(0.92)								
4.	PGIS authentic	4.63 (0.54)	−0.03	0.09	0.83**	(0.95)							
5.	MBIE involve	5.58 (0.54)	0.01	0.10	0.60**	0.53**	(0.91)						
6.	MBIE influence	5.18 (0.80)	0.02	0.08	0.39**	0.38**	0.69**	(0.89)					
7.	Share resource	30.55 (5.88)	0.10	−0.10	0.06	0.06	0.09	0.15*	–				
8.	Age	40.16 (13.69)	−0.02	0.14*	−0.06	−0.03	−0.00	−0.06	−0.04	–			
9.	Gender	1.51 (0.50)	0.09	0.11	0.08	0.07	0.09	0.11	0.06	0.04	–		
10.	Ethnicity	0.83 (0.38)	−0.01	0.02	0.17**	0.14*	0.08	0.02	−0.01	0.16**	−0.01	–	
11.	Employed	0.74 (0.44)	−0.02	−0.10	0.10	0.05	−0.03	0.04	−0.10	−0.20**	−0.09	−0.06	-
12.	Exper. teams	5.83 (1.50)	0.01	−0.02	0.21**	0.18**	0.17**	0.12*	−0.07	0.02	0.08	0.09	0.34**

Scale reliabilities are shown in parentheses along the diagonal. Div. statement = Age diversity statement (0 = no statement, 1 = statement). Nr older = Number of older individuals selected into the team. PGIS member = group membership. PGIS authentic = room for authenticity. MBIE involve = work group involvement. MBIE influence = influence in decision making. Share resource = share of 100 Euro team bonus allocated to older team member. Age = participant age (in years). Gender = participant gender (1 = male, 2 = female). Ethnicity = participant ethnicity (0 = Non-White/Caucasian, 1 = White/Caucasian). Employed = participant employment status (0 = currently not employed, 1 = currently employed). Exper. teams = participants’ experience of work in age diverse teams. * *p* < 0.05. ** *p* < 0.01.

**Table 2:** Overall effects of age diversity and age diversity and inclusion statements on selecting older individuals into teams (diversity) and inclusive behavior toward older teammates (inclusion).

	No statement		Diversity statement		Diversity & inclusion statement				
	*M*	SE	*M*	SE	*M*	SE	*F*	*p*	Partial η^2^
**Study 1**									
*Diversity*									
Nr older teammates	0.98	0.04	1.09	0.04	–	–	3.770	0.053	0.013
*Inclusion*									
PGIS member	4.70	0.04	4.69	0.04	–	–	0.066	0.797	0.000
PGIS authentic	4.65	0.05	4.61	0.05	–	–	0.234	0.629	0.001
MBIE involve	5.58	0.05	5.58	0.05	–	–	0.002	0.963	0.000
MBIE influence	5.18	0.07	5.19	0.07	–	–	0.027	0.870	0.000
Resource share	30.03	0.49	31.09	0.50	–	–	2.308	0.130	0.008
**Study 2**									
*Diversity*									
Nr older teammates	0.85	0.05	1.09	0.03	1.05	0.03	9.941	0.001	0.041
*Inclusion*									
PGIS member	5.54	0.06	5.63	0.04	5.60	0.04	0.956	0.385	0.004
PGIS authentic	5.44	0.07	5.50	0.05	5.49	0.05	0.314	0.731	0.001
MBIE involve	5.37	0.07	5.49	0.05	5.47	0.05	1.397	0.248	0.006
MBIE influence	5.06	0.08	5.14	0.06	5.12	0.06	0.382	0.683	0.002
Resource share	29.16	0.59	31.04	0.40	30.94	0.40	3.929	0.020	0.017

Estimated marginal means are shown. Nr older = Number of older individuals selected into the team. PGIS member = group membership. PGIS authentic = room for authenticity. MBIE involve = work group involvement. MBIE influence = influence in decision making. Share resource = share of 100 Euro team bonus allocated to older team member.

To test Hypothesis 2, we conducted five ANCOVA (see above), each with one indicator of inclusion as the dependent variable. Results showed no significant differences between conditions for all five indicators (see Table 2, upper half). Thus, across all indicators, participants exposed to the age diversity statement did not report more frequent intentions to engage in inclusive behaviors toward the older teammate compared to participants who were not exposed to the statement.

### Discussion

The results of Study 1 show that the age diversity statement increases the representation of older employees, which is in line with previous research. However, age diversity statements did not increase inclusion. Participants choose more older individuals into their team after having been exposed to an age diversity statement, but they are not more inclusive towards older individuals on their team. This could be due to the content of the statement used in this study, which referred to “diversity” without explicitly mentioning “inclusion.” We tested this possibility in Study 2 by explicitly manipulating the target of the statement (diversity vs. diversity and inclusion).

In addition, some unintended signals of diversity statements (Dover et al., 2020) could explain the absence of impact on inclusion, as reported in Study 1. Diversity statements help organizations promote diversity and inclusion (Jansen et al., 2021), but people become skeptical since they are often used as mere reputational instruments (Point and Singh, 2003; Wang et al., 2023). Thus, diversity statements do not always signal the organization’s genuine commitment to diversity (Dover et al., 2020; Wilton et al., 2020) but may be used instead to boost organizational image and reputation (Toma et al., 2023). To assess this, we manipulated the underlying organizational motivation for diversity and inclusion in Study 2.

In this new study, we told participants that the organizational motivation for diversity and inclusion was either true change or a reputation boost. In the change condition, we hypothesized that age-diversity statements would lead participants to choose more age-diverse teams and show more inclusive behaviors (compared to the control condition). In the reputation condition, we hypothesized that age-diversity statements would lead participants to choose more age-diverse teams (compared to the control condition) but that they may not necessarily behave more inclusively.

## Study 2

### Method

#### Participants

We recruited 518 U.S. and U.K. residents through Prolific. Participants were paid £1.20 for a study we expected to take 8 min, corresponding to an hourly wage of £9. After excluding respondents who failed the attention check (*n* = 31) and who did not indicate their gender (*n* = 9), the final sample consisted of 478 participants (mean age 39.02, SD = 12.83, 49.4% men). The majority were employed (60.7% full-time, 19.0% part-time), and the remaining 20.3% were unemployed.

#### Procedure

The experiment had a 2 (age diversity statement: diversity or diversity and inclusion) x 2 (organizational motive: reputation or change) between-subjects design, with age diversity statement and organizational motive as between-subjects factors. Moreover, we added a control condition in which participants were not shown a diversity statement. As in Study 1, participants had to create a team of three persons to complete the project by selecting two teammates from a list of four potential teammates who varied in age and gender. Before choosing their teammates and indicating their inclusive behaviors towards an older teammate, participants saw a general age diversity statement (like in Study 1), followed by a specific one, which varied according to the condition. In the reputation condition, participants read, “The reason the company introduced this policy is to improve its reputation on age diversity.” In the change condition, participants read: “The reason the company introduced this policy is to improve the representation of their older employees.” The target of the age diversity statement was manipulated in the next phrase. In the condition targeting diversity, the phrase read “Thus, the company encourages you to work in teams in which older employees are well represented,” while in the condition targeting diversity and inclusion, it read “Thus, the company encourages you to work in teams in which older employees are well represented and feel included”.

As in Study 1, after choosing their teammates, participants were informed that another older colleague joined their team. Subsequently, they indicated how much they intended to engage in inclusive behaviors toward this new team member. At the end of the survey, participants answered demographic questions.

#### Measures

We used the same measures of representation and inclusion toward older teammates and the same control variables as in Study 1 (see text footnote 1).

### Results

Correlations, descriptive statistics, and reliabilities of the main study variables are presented in Table 3. To examine the effects of the age diversity statement’s target on team age diversity, we conducted an ANCOVA with the number of selected older teammates as an outcome, age diversity statement condition (diversity vs. diversity and inclusion vs. no statement) as a predictor, and participants’ age, gender, ethnicity, employment status and experience of work in age-diverse teams as controls. Descriptive statistics per condition and results are displayed in Table 2 (lower half), showing the main effect of the age diversity statement. Follow-up pair-wise comparisons using Sidak adjustments reveal that both statements increased the number of selected older teammates such that participants were more likely to select an older individual into their team with an age diversity statement and an age diversity and inclusion statement in place, compared to when there was no age diversity statement. There were no differences between the two statements.

**Table 3:** Study 2: means, standard deviations, reliabilities and correlations between study variables.

		Mean (*SD*)	1	2	3	4	5	6	7	8	9	10	11	12
1.	Div. statement	1.22 (0.74)	–											
2.	Org. motive	1.21 (0.74)	0.63**	–										
3.	Nr older	1.03 (0.45)	0.15*	0.14**	–									
4.	PGIS member	5.60 (0.51)	0.04	0.10*	0.16**	(0.90)								
5.	PGIS authentic	5.48 (0.61)	0.03	0.08	0.11*	0.74**	(0.94)							
6.	MBIE involve	5.46 (0.62)	0.05	0.10*	0.17**	0.73**	0.69**	(0.93)						
7.	MBIE influence	5.12 (0.75)	0.03	0.08	0.09	0.53**	0.60**	0.75**	(0.87)					
8.	Share resource	30.65 (5.55)	0.09*	0.07	−0.01	0.08	0.09*	0.19**	0.23**	–				
9.	Age	39.02 (12.83)	−0.06	−0.08	0.21**	0.13**	0.07	0.10*	−0.32	−0.05	–			
10.	Gender	1.51 (0.50)	−0.02	0.05	0.16**	0.08	0.09	15**	0.10*	−0.02	0.01	–		
11.	Ethnicity	0.85 (0.36)	−0.12**	−0.06	0.03	0.05	0.09*	0.10*	0.10*	−0.04	0.19*	0.05	–	
12.	Employed	0.80 (0.40)	−0.02	0.02	−0.10*	−0.08	0.00	−0.09	−0.02	0.03	−0.20**	0.00	−0.03	–
13.	Exper. teams	5.79 (1.49)	0.06	0.09	0.01	0.13**	0.20**	0.15**	0.15**	−0.01*	0.16**	−0.02	0.06	0.35**

Scale reliabilities are shown in parentheses along the diagonal. Div. statement = Age diversity statement (0 = no statement, 1 = diversity statement, 2 = diversity and inclusion statement). Org. motive for diversity (0 = no statement, 1 = reputation, 2 = change). Nr older = Number of older individuals selected into the team. PGIS member = group membership. PGIS authentic = room for authenticity. MBIE involve = work group involvement. MBIE influence = influence in decision making. Share resource = share of 100 Euro team bonus allocated to older team member. Age = participant age (in years). Gender = participant gender (1 = male, 2 = female). Ethnicity = participant ethnicity (0 = Non-White/Caucasian, 1 = White/Caucasian). Employed = participant employment status (0 = currently not employed, 1 = currently employed). Exper. teams = participants’ experience of work in age diverse teams. * *p* < 0.05. ** *p* < 0.01.

To examine the effects of the age diversity statement’s target on age-inclusive behaviors, we conducted the same analyses as above, with the five inclusion indicators as dependent variables. The results of the five ANCOVAs reported no significant differences between conditions for all indicators except for resource sharing (see Table 2, lower half). Pairwise follow-up comparisons showed that in both statement conditions, participants allocated slightly more money and thus a more equal share of the bonus to the older teammate, compared to when there was no statement. Thus, both diversity statements increased age-inclusive behaviors when distributing financial resources but did not affect other inclusive behaviors.

To examine the moderating effect of the organizational motivation on selecting older teammates, separately for the diversity statement and the diversity and inclusion statement conditions, we conducted two ANCOVAs. Descriptive statistics per condition and results are displayed in Table 4. First, when looking at the diversity statement condition, results showed that selection rates differed between the three conditions (reputation vs. change vs. control) (see Table 4, upper half). Follow-up pairwise comparisons using Sidak adjustments indicated that compared to the control condition, both the reputation and the change motive increased the number of older individuals in teams. Furthermore, there were no differences between the two organizational motives, *p* = 0.794. Second, when looking at the diversity and inclusion statement condition, results showed that rates differed between the three conditions (see Table 4, lower half). Follow-up pairwise comparisons using Sidak adjustments reported that compared to the control condition, both using the reputation and the change motive increased the number of older individuals in teams. There were no differences between the two motivations. In sum, we found no evidence that organizational motives moderated the positive effects of statements targeting diversity and those targeting diversity and inclusion on the representation of older individuals in teams. Statements were equally effective, independently of the organizational motivation.

**Table 4:** Study 2: effects of organizational motivation for age diversity (upper half) and age diversity and inclusion (lower half) on selecting older individuals into teams (diversity) and inclusive behavior toward older teammates (inclusion).

	Diversity: reputation		Diversity: change		No statement				
	*M*	SE	*M*	SE	M	SE	*F*	*p*	Partial η^2^
*Diversity*									
Nr older teammates	1.12	0.05	1.06	0.05	0.86	0.05	8.960	0.001	0.061
*Inclusion*									
PGIS member	5.64	0.05	5.61	0.05	5.54	0.06	0.996	0.371	0.007
PGIS authentic	5.54	0.06	5.47	0.06	5.44	0.07	0.657	0.519	0.005
MBIE involve	5.51	0.06	5.49	0.06	5.37	0.07	1.153	0.219	0.011
MBIE influence	5.19	0.08	5.10	0.08	5.06	0.08	0.852	0.428	0.006
Resource share	31.30	0.57	30.71	0.59	29.26	0.61	3.132	0.045	0.022

	Div.& Incl.: reputation		Div. & Incl.: change		No statement				
*Diversity*									
Nr older teammates	1.02	0.05	1.08	0.05	0.85	0.05	6.453	0.002	0.045
*Inclusion*									
PGIS member	5.51	0.06	5.69	0.06	5.54	0.06	3.492	0.032	0.025
PGIS authentic	5.38	0.06	5.58	0.06	5.43	0.07	3.030	0.050	0.021
MBIE involve	5.39	0.06	5.53	0.06	5.36	0.07	2.014	0.135	0.014
MBIE influence	5.02	0.08	5.22	0.08	5.05	0.08	2.036	0.133	0.015
Resource share	31.25	0.62	30.65	0.62	29.18	0.65	2.750	0.066	0.020

Estimated marginal means are shown. Nr older = Number of older individuals selected into the team. PGIS member = group membership. PGIS authentic = room for authenticity. MBIE involve = work group involvement. MBIE influence = influence in decision making. Share resource = share of 100 Euro team bonus allocated to older team member.

To examine the moderating effect of organizational motivation on age-inclusive behaviors, we conducted the same analyses as above, with the five inclusion indicators as outcome variables. First, when looking at the diversity statement condition (see Table 4, upper half), the results of the five ANCOVAs revealed that there were no differences between the three conditions (reputation vs. change vs. control) for the PGIS and MBIE indicators. The effect for resource sharing was significant and follow-up pairwise comparisons indicated that participants gave slightly more money to the older teammate in the diversity motivated by reputation condition. No other differences emerged.

Second, when looking at the diversity and inclusion statement condition (see Table 4, lower half), results of the five ANCOVAs showed a significant difference between conditions for treating the older individual as belonging to the group and letting the individual be authentic. Results of the follow-up pairwise comparisons revealed that participants were more likely to display these inclusive behaviors when the diversity and inclusion statement was motivated by change compared to reputation, while there was no difference between the reputation and control conditions. No other differences emerged.

### Discussion

Replicating and extending Study 1, diversity statements, regardless of whether they target diversity or diversity and inclusion, and irrespective of the organizational motivation, lead participants to choose more older team members compared to the control condition.

The results were more nuanced and in line with our expectations on inclusive behaviors. Regarding the impact of diversity statements, we found similar results to those of Study 1. Diversity statements, regardless of whether they target diversity or diversity and inclusion, do not influence inclusive behaviors, except for the resource allocation measure. Unlike in Study 1, participants in Study 2 allocated more money to the new, older employee in the two diversity statement conditions compared with the control condition.

Notably, the effects on inclusion depend on organizational motivation, but only when the diversity statements target both diversity and inclusion. When the diversity statement targets diversity only, inclusive behavior is not influenced by organizational motivation and does not differ from the control condition. This replicates what we found in Study 1. The only exception regards the resource allocation measure, as participants allocate slightly more money to the older team member when the motivation is reputation, compared to the control condition; but not when the motivation is true change. While there was no effect on resource allocation in Study 1, indicating that this finding may be less robust, this pattern remains unexpected. It could potentially be explained by the fact that participants were driven to compensate older teammates in financial terms when the organizational commitment to inclusion was perceived as superficial (i.e., when the diversity statement only targeted representation and when the organization’s motive was reputation).

However, in line with our expectations, when the diversity statement targets both diversity and inclusion, we find that participants intend to be more inclusive if the motivation is true change compared to reputation. This effect is significant for providing belongingness and leaving room for authenticity but not significant for group involvement and providing influence in decision-making. Thus, while diversity and inclusion statements that reflect organizational motivation for true change can foster inclusion, their effect may be limited to certain behaviors.

## General discussion

In two studies, we found consistent evidence for the assumption that diversity statements increase the representation of older employees in teams, but that they do not trigger inclusive behaviors alone. These results have important theoretical and practical implications. At the theoretical level, we contribute to the debate about whether diversity statements are useful to create more diverse and inclusive teams. While organizations use diversity statements to publicly signal that they value diversity (Wang et al., 2023), employees may not behave as expected (Leslie, 2019; Dover et al., 2020). Some studies suggest that diversity statements that are not accompanied by evidence about the results (Wilton et al., 2020; De Cock et al., 2024) or about the organization’s motivation (Cole et al., 2022) can lead to mixed or negative outcomes. We find that diversity statements can be nevertheless effective and lead to more age diversity also when the organization’s motivation is unknown reproducing earlier findings from a context where team performance was real and financially incentivized (De Saint Priest and Krings, 2024), However, we find people are not more inclusive toward older team members. This paradoxical effect may be due to moral licensing (Effron and Conway, 2015), as people may perceive their choice for older team members as a moral behavior that ‘liberates’ them from inclusion towards the new member. Only when the statement stresses both diversity and inclusion and when the organization explicitly communicates its commitment to true change, we find positive effects for some inclusive behaviors (e.g., creating feelings of belongingness) but not for others (e.g., providing opportunities to influence decisions).

These findings also have important practical implications. It suggests that the diversity statement’s content and the underlying organizational motivation matter for diversity and inclusion. Because organizations’ goal is to create inclusive work climates, having broad diversity statements without explicit reference to inclusion may not be enough. In addition, it is key for organizations to clearly communicate their motivation to create change in the workforce. Without this information, employees may be skeptical and infer that organizations use diversity statements for reputational concerns. It is important to note that this effect might not be limited to age diversity. The present results might be relevant for other diversity dimensions, such as gender, race, sexual orientation, disability, etc., suggesting that the massive use of diversity statements may lead to paradoxical and unintended effects.

This research has some limitations that should be addressed in future studies. In addition to being based on hypothetical scenarios and using samples limited in size, another important limitation is that we did not directly examine the underlying mechanisms explaining the impact of diversity statements on diversity and inclusion. While previous research suggests that diversity statements may increase representation primarily because they clearly signal what is desirable in the organization (De Saint Priest and Krings, 2024), this process may further depend on the organization’s motives. We argued that the organizational motivation for true change can be a powerful driver of employees’ motivation for diversity and inclusion, which translates into concrete behaviors for diversity and inclusion. We also suspect that the motivation for reputation triggers compliance, perhaps coupled with a moral licensing effect. Future research is needed to test these mechanisms and further comprehend the complex interplay between diversity and inclusion.

In conclusion, age diversity statements increase the representation of older employees in teams but may not necessarily promote inclusion. Inclusive behaviors require the organization to be explicit about inclusion and its motivation to achieve change.

## Data availability statement

The raw data supporting the conclusions of this article will be made available by the authors, without undue reservation.

## Ethics statement

The studies involving humans were approved by Ethics Committee, LABEX, Faculty of Business and Economics (HEC), University of Lausanne. The studies were conducted in accordance with the local legislation and institutional requirements. The participants provided their written informed consent to participate in this study.

## Author contributions

ODSP: Formal analysis, Methodology, Writing – original draft, Writing – review & editing. FK: Conceptualization, Formal analysis, Methodology, Writing - original draft, Writing – review & editing. CT: Conceptualization, Methodology, Writing – original draft, Writing – review & editing.

## Footnotes

^1^Excluding the control variables from the analyses did not affect the pattern of results.
